# Synthesis of surface-modified iron oxide nanocrystals using supercritical carbon dioxide as the reaction field

**DOI:** 10.1039/d1ra08580h

**Published:** 2022-03-11

**Authors:** Yasuhiko Orita, Keito Kariya, Thossaporn Wijakmatee, Yusuke Shimoyama

**Affiliations:** Department of Chemical Science and Engineering, Tokyo Institute of Technology 2-12-1 S1-33, Ookayama Meguro-ku Tokyo 152-8550 Japan orita.y.aa@m.titech.ac.jp

## Abstract

In the synthesis of surface-modified nanocrystals (NCs), a simple and green chemistry approach to reduce liquid waste, particularly a solventless process, has been desired. In this study, we applied the supercritical CO_2_ technology, which is an excellent solventless process, to the synthesis of surface-modified iron oxide NCs. The synthesis was performed at 30.0 ± 0.8 MPa of CO_2_, 18 h and 100 °C, where iron(iii) acetylacetonate, pure water and decanoic acid were used as starting materials. As a result, the supercritical CO_2_ medium gave the NCs of α-Fe_2_O_3_ and γ-Fe_2_O_3_ with unimodal size distribution, where the mean size was 7.8 ± 2.0 nm. In addition, they were self-assembled on the TEM substrate and the mean nearest-neighbor spacing was close to the chain length of decanoic acid. Furthermore, FT-IR and TG analyses indicate that decanoic acid chemically attaches to the surface of iron oxide NCs that are dispersed in cyclohexane. These results suggest that the supercritical CO_2_ medium could be the new appealing reaction field to fabricate densely modified NCs without liquid waste.

## Abstract 2

We applied the supercritical CO_2_ technology, which is an excellent solventless process, to the synthesis of surface-modified iron oxide nanocrystals.

## Graphical abstract

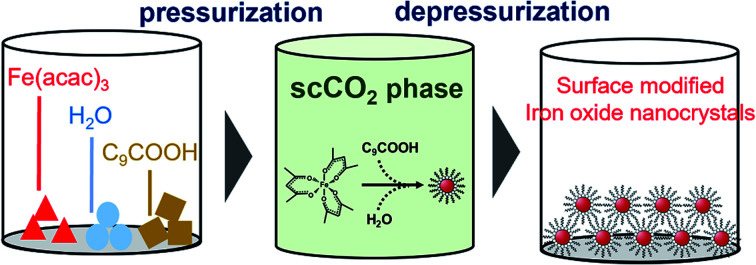

## 1. Introduction

Surface-modified metal oxide nanocrystals (NCs) have attracted considerable interest in recent years.^1^ Although the aggregation of NCs due to their high surface energy is a serious problem for practical utilization such as thin film fabrication,^2^ patterning on substrates^3^ and drug carriers,^4^ surface modification largely reduces the surface energy and improves their dispersibility to solvent, thus preventing the aggregation.^5^ In addition, surface modification at the synthesis stage allows the inhibition of not only aggregation but also the control of the size and shape of NCs that strongly affects their physicochemical properties.^6,7^ These appealing effects of surface modification have promoted the research on the direct-synthesis methods for surface-modified NCs.^7–10^

The surface-modified NCs has been typically synthesized *via* wet-based methods such as sol–gel,^8^ hot-injection,^9^ heat-up^11^ and hydrothermal^10^ methods because the solvent is required to dissolve precursors and surfactants uniformly and to control the reaction of them.^12^ However, wet-based methods cause a large amount of liquid waste for the synthesis and washing,^9–11^ where the disposal and the regeneration cost of them is known as the critical issue. However, the synthesis in supercritical carbon dioxide (CO_2_) can be new appealing candidate to fabricate surface-modified NCs. Supercritical CO_2_ has unique properties such as high solubility of the metal organic precursor^13^ and diffusivity, which allows the formation of the homogenous phase,^14^ while the synthesis in supercritical CO_2_ is substantially the solventless reaction process. In addition, supercritical CO_2_ can be used not only for synthesis but also as a washing and drying solvent for the particle production.^14,15^ These characteristics allow the simple fabrication process without liquid waste; thus, the supercritical CO_2_ medium has been applied for the synthesis of inorganic materials such as metal oxides,^16^ metal hydroxides^15^ and metal sulfates.^17^ However, in the most of case, the products were observed as aggregates or the submicron-sized particles after the synthesis in supercritical CO_2_.^16,18^ This is probably due to the characteristic of supercritical CO_2_ that is non-polar and low viscous solvent. The non-polar properties of supercritical CO_2_ have low compatibility with the metal oxide surface (generally is hydrophilic), which typically results in the accelerated aggregation. Furthermore, low viscous properties generally lead to the vigorous Brownian movement of particles, which also accelerate the aggregation. The introduction of an organic surfactant to the synthesis in supercritical CO_2_ is expected to overcome this serious problem because surface modification can change the surface properties of metal oxides from hydrophilic to hydrophobic that is compatible to supercritical CO_2_.^19^ In addition, the reduction of surface energy and stearic repulsion between particles can be achieved by surface modification.^4,19^ Furthermore, supercritical CO_2_ has high ability to dissolve the organic surfactant that is typically used for the synthesis of surface-modified NCs such as saturated and unsaturated fatty acids.^12,20–22^ Therefore, supercritical CO_2_ with the organic surfactant has the potential to directly synthesize the surface-modified NCs with good monodispersity. However, the report concerned with the synthesis in supercritical CO_2_ is limited to the metal oxide without surface modification;^15,18,23^ thus, the direct synthesis of surface-modified NCs in supercritical CO_2_ was attempted in this study.

In this study, we report a novel simple synthesis using supercritical CO_2_ as a reaction medium for surface-modified NCs. As a model material, we chose iron oxide that is applied as the catalyst,^24^ drug carrier^25^ and in a magnetic recording device^26^ due to its appealing catalytic and magnetic properties. Iron(iii) acetylacetonate and decanoic acid of fatty acids were used as the precursor and surfactant, respectively, since they are highly stable and commercially available reagents, which make them desirable candidates for the synthesis. Herein, the hydrolysis or thermolysis of the iron precursor is a popular reaction to synthesize iron oxide NCs for conventional hydrothermal and heat-up methods.^11,19^ The thermolysis reaction of the iron precursor requires a high temperature above 300 °C, where such a severe environment easily produces the byproduct sourced from the thermal decomposition of a high boiling solvent (commonly dibenzyl ether).^27^ On the other hand, the hydrolysis reaction of the iron precursor easily proceed under 100 °C and yield the well crystalline nanoparticles;^28^ therefore, a small amount of water was used as a starting material to utilize the hydrolysis reaction for the synthesis of iron oxide NCs in this study.

## 2. Experimental

### 2.1 Materials

Iron(iii) acetylacetonate [Fe(acac)_3_] (purity > 99%) and decanoic acid (purity > 98.0%) were purchased from Wako Pure Chemical Industries, Ltd. CO_2_ (purity > 99.9%), nitrogen (N_2_, purity > 99.95%) and ultra-high pressure N_2_ (purity > 99.95%) were supplied by Fujii Bussan Co., Ltd. Ultra-pure water was prepared using a Direct-Q UV3 Water Purification System supplied by EMD Millipore Corp., and the resistivity was confirmed to be 18.2 MΩ cm.

### 2.2 Synthesis

**Fig. 1:**
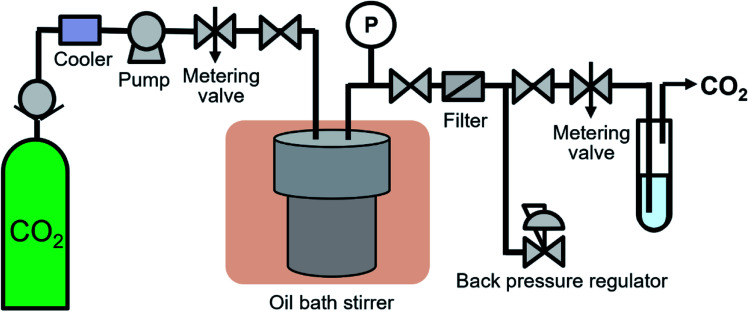
Experimental apparatus.

The high-pressure system shown in Fig. 1 was developed to synthesize surface-modified NCs in supercritical CO_2_. 0.53 g of Fe(acac)_3_, 1.29 g of decanoic acid and 0.45 g of water were transferred to 76 mL volume of a reaction vessel (TSC-CO_2_-008; Taiatsu Glass Corp.). The molality of Fe(acac)_3_, decanoic acid and water were 0.03, 0.15 and 0.50 mol kg^−1^, respectively, which were calculated using the volume of the vessel and CO_2_ density under the given reaction conditions. The mixture was stirred for 1 min under ambient conditions. Subsequently, CO_2_ flowed under 0.5 MPa for 1 min to displace the air in the vessel and liquified CO_2_ was introduced into the vessel using the HPLC pump (PU-4386; JASCO Co., Ltd.) until reaching 6.6 to 7.0 MPa. After reaching the appropriate pressure, the vessel was sunk in the oil bath stirrer, which resulted in the target temperature of 100 °C and the pressure of 30.0 ± 0.8 MPa. The vessel was left in the oil bath stirrer for 18 h, while the enclosed materials were vigorously stirred. After the time passed, the vessel was pulled up and was depressurized at a rate of approximately 0.5 MPa min^−1^ using a metering valve (1315G2Y; HOKE Inc.). After depressurization, the vessel was quenched in a water bath at room temperature.

The experiments were also performed at N_2_ of 30.0 MPa and at N_2_ atmosphere as reference controls. In the experiment at 30.0 MPa, N_2_ was introduced into the vessel using an ultra-high pressure N_2_ cylinder and the high-pressure system shown in Fig. 1 until reaching 22.1 MPa. The cylinder was connected to the middle point between the pump and the metering valve. After reaching the pressure, the vessel was sunk in the oil bath stirrer, which resulted in the target pressure of 30.0 ± 0.3 MPa. In the experiment at N_2_ atmosphere, the vessel was purged by N_2_. Subsequently, the inlet and outlet valves were closed and the vessel was sunk in the oil bath stirrer. In both cases, other conditions and procedures were same as the case using supercritical CO_2_.

The products were collected by rinsing the reaction vessel successively with a cyclohexane/ethanol mixture whose volume ratio was 1 : 4. The products were centrifuged and washed with a mixture of cyclohexane and ethanol (1 : 4) to eliminate the unreacted precursor and surfactant. The solid products were dried in a vacuum oven at room temperature for 24 h.

### 2.3 Characterization

The particle yield *Y* was defined as follows.1
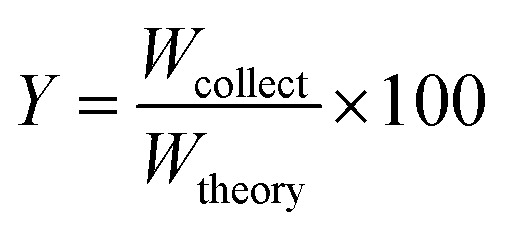
where *W*_collect_ is the weight of final products after washing and drying and *W*_theory_ is the maximum particle weight of Fe_2_O_3_ when the reaction is complete. X-ray diffraction patterns of the products were obtained *via* X-ray diffraction (XRD) (MiniFlex600-C; Rigaku Corp.) using Cu Kα radiation. The crystallite size was evaluated using the Scherrer equation, where 0.90 of shape factor was used. The products were observed by transmission electron microscopy (TEM) (H-7650; Hitachi Corp.) operated at 100 kV. High resolution TEM (HR-TEM) images and electron diffraction pattern of particle colony were acquired using a JEOL JEM-2010F electron microscope operated at 200 kV. The solid products for TEM and HR-TEM analysis were dispersed in cyclohexane before transferred onto the copper grids with a organic membrane. The mean size, with their standard deviation, of the particles was determined by observing approximately 200 particles for TEM analysis. Thermogravimetric analysis was performed from room temperature to 600 °C at a ramp rate of 10 °C min^−1^ and at N_2_ atmosphere using a thermogravimetric analyzer (TGA) (TGA-50; Shimadzu Corp.). The absorbed state of an organic surfactant on the iron oxide surface was investigated using a Fourier transform infrared spectrometer (FT-IR) (FT-IR4100; JASCO Co., Ltd.).

## 3. Results and discussion

Table 1 shows experimental conditions and results. The particle yields were less than 1% at 30.0 MPa of CO_2_ without water, at N_2_ atmosphere with water and at 30.0 MPa of N_2_ with water, as shown in products 1 to 3 from Table 1. On the other hand, they went beyond 50% regardless of the existence of decanoic acid at CO_2_ of 30.0 MPa with water. These results clearly indicate that the addition of water to the supercritical CO_2_ medium accelerated the particle formation. The thermolysis rate of Fe(acac)_3_ is known to be very small, and the calculated conversion under the reaction conditions (100 °C, 18 h) was negligible based on the report from G. Beech and R. Lintonbon.^29^ Therefore, the hydrolysis reaction should be key role to form iron oxide. However, water is not miscible with Fe(acac)_3_; in addition, high pressure N_2_ typically has low solubility due to its low density (corresponding to 234 kg m^−3^ at 30.0 MPa and 100 °C).^30^ This suggests that Fe(acac)_3_ and water face the problem of diffusion limit at N_2_ atmosphere and at N_2_ of 30.0 MPa. In contrast, supercritical CO_2_ (with high density of 662 kg m^−3^) has high solubility of Fe(acac)_3_ (ref. 13) and water^31^ and diffusibility; thus, the hydrolysis reaction of the Fe(acac)_3_ molecule could be accelerated in supercritical CO_2_, which plausibly promotes the particle formation. Herein, focusing on the difference of particle yields between products 4 and 5, the yield increased from 52 to 76% by introducing the decanoic acid into the supercritical CO_2_ medium. The presence of decanoic acid on the particle surface possibly raises the weight of particle and the yield. The modification state of the particle surface by decanoic acid is discussed later.

**Table 1:** Experimental conditions and results

Products	Fe(acac)_3_ (g)	Water (g)	Decanoic acid (g)	Atmosphere	Pressure (MPa)	Yield (%)
1	0.530	0.00	1.29	CO_2_	30.0	<1
2	0.530	0.45	1.29	N_2_	0.1	<1
3	0.530	0.45	1.29	N_2_	30.0	<1
4	0.530	0.45	0.00	CO_2_	30.0	52
5	0.530	0.45	1.29	CO_2_	30.0	76

**Fig. 2:**
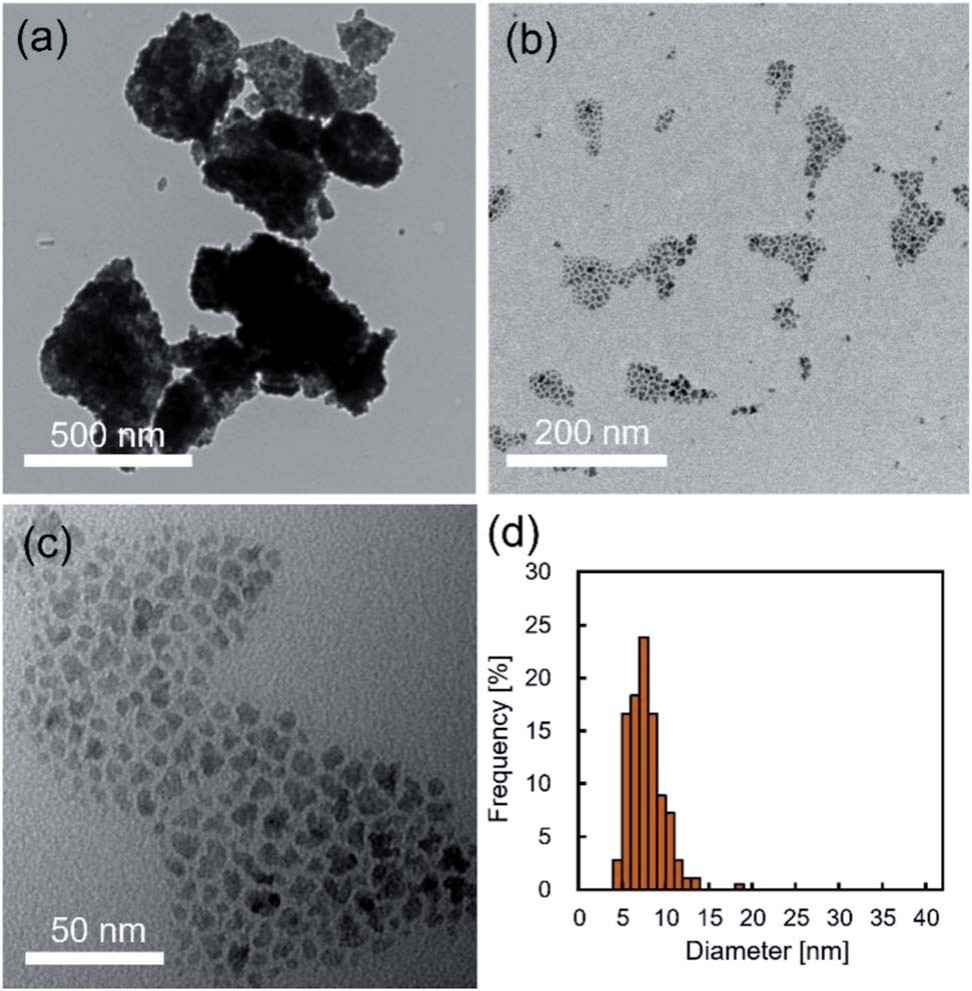
(a–c) The typical TEM images of NCs synthesized in supercritical CO_2_ (a) without decanoic acid and (b and c) with decanoic acid. (d) Size distribution histogram of NCs synthesized in supercritical CO_2_ with decanoic acid.

Fig. 2a–c show typical TEM images of the NCs prepared in supercritical CO_2_. The products synthesized without decanoic acid showed aggregation state (Fig. 2a), while the products that were synthesized with decanoic acid self-assembled on the TEM substrate (Fig. 2b and c), where the mean nearest-neighbor spacing was calculated to be 2.4 ± 0.7 nm by observing about 100 spacing. The decanoic acid has a carbon chain length of 1.4 nm, thus, the distance between self-assembled NCs can be seen as approximately 2.8 nm.^19^ This theoretical distance was somewhat closed to our observed distance of 2.4 ± 0.7 nm, indicating that the surface of obtained NCs was modified enough densely to achieve stearic hindrance. In addition, the supercritical CO_2_ medium with decanoic acid gave the NCs with unimodal and narrow size distribution, as shown in Fig. 2d, and the mean size of NCs was 7.8 ± 2.0 nm, which supported that the aggregation of NCs did not occur. In general, surface modification by surfactant, such as decanoic acid, enhances stearic repulsion force between particles, as shown in Fig. 2c, and reduces surface energy.^4^ In addition, it can change the surface properties of metal oxide NCs from hydrophilic to hydrophobic, which is compatible to supercritical CO_2_.^19^ These effects typically allow the inhibition of the aggregation of NCs. Therefore, it can be concluded that the addition of decanoic acid to supercritical CO_2_ effectively modifies the surface of NCs, which allows the inhibition of the aggregation in the synthetic field.

**Fig. 3:**
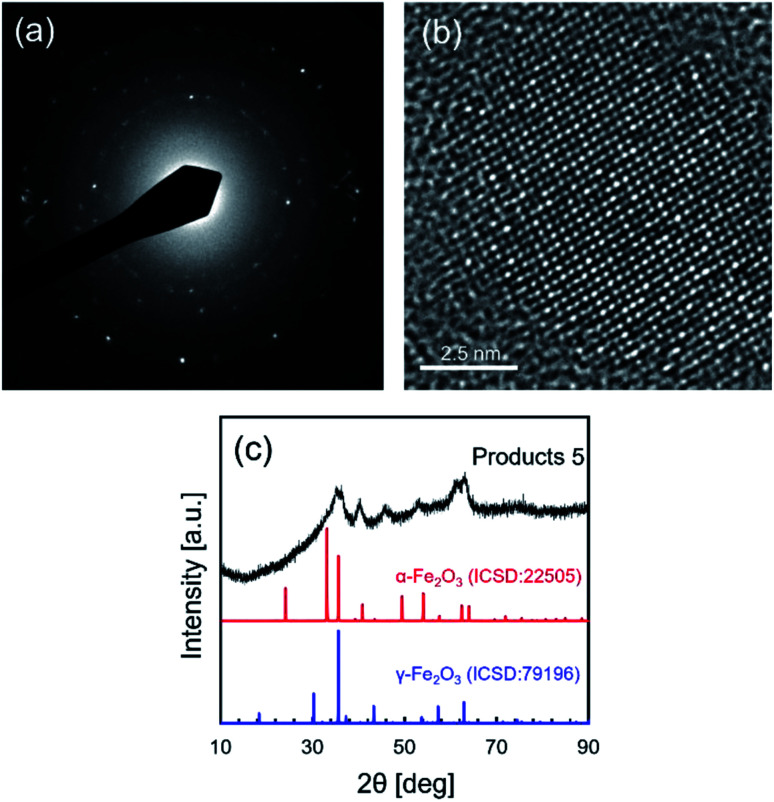
(a) The electron diffraction pattern, (b) the high resolution TEM image and (c) the XRD pattern of NCs synthesized in supercritical CO_2_ with decanoic acid (products 5 listed in Table 1).

High resolution TEM, TEM electron diffraction and XRD analysis were performed to analyze the crystallinity and crystal structure of NCs synthesized in supercritical CO_2_ with decanoic acid, as shown in Fig. 3a–c. In the electron diffraction pattern, the scattered diffraction spot and the Debye–Scherrer ring were observed, indicating that the obtained NCs have good crystallinity. The good crystallinity of NCs was further confirmed by observing the atomic arrangement from the HR-TEM image shown in Fig. 3b. In addition, the crystallite size (calculated from the XRD peak at 40.2° in Fig. 3c) was 6.0 nm that is somewhat close to the size obtained by TEM, suggesting that the as-synthesized NCs have single crystallinity. However, the as-prepared NCs showed a broad XRD pattern (Fig. 3c), which may be because of the very small particle size and the low volume portion of iron oxide due to the core–shell structure of the iron oxide core and decanoic acid shell. A small particle size (corresponding to small crystallite size) typically broadens the widths of PXRD peaks. In addition, as stated in the previous paragraph, Fig. 2c evidentially shows that iron oxide NCs is densely covered with decanoic acid enough to bring the spacing between particles, supporting the formation of the core–shell structure.^1^ For the core–shell structure, the volume portion of iron oxide is calculated to be 39.8% using the size of iron oxide NCs and the carbon chain length of decanoic acid. This low volume portion causes the reduction of the actual crystallite volume for PXRD measurement, which results in the decreasing intensity. Therefore, the small particle size and low volume portion of iron oxide NCs plausibly result in the broad XRD pattern. Table 2 lists the lattice spacings calculated by the electron diffraction pattern (shown in Fig. 3a) of obtained NCs, where the lattice spacings were assigned to the crystal structure of α-Fe_2_O_3_ (ICSD: 22505) and γ-Fe_2_O_3_ (ICSD: 79196).

**Table 2:** Lattice spacings and assigned structures of NCs synthesized in supercritical CO_2_ with decanoic acid

No.	Lattice spacing^a^ [nm]	Assigned structure^b^	
1	0.295		γ-Fe_2_O_3_
2	0.249	α-Fe_2_O_3_	γ-Fe_2_O_3_
3	0.224	α-Fe_2_O_3_	
4	0.203		γ-Fe_2_O_3_
5	0.168	α-Fe_2_O_3_	γ-Fe_2_O_3_
6	0.148	α-Fe_2_O_3_	γ-Fe_2_O_3_

a Lattice spacings were calculated by TEM electron diffraction pattern.

b The lattice spacings were assigned to the crystal structure of α-Fe_2_O_3_ (ICSD: 22505) and γ-Fe_2_O_3_ (ICSD: 79196).

**Fig. 4:**
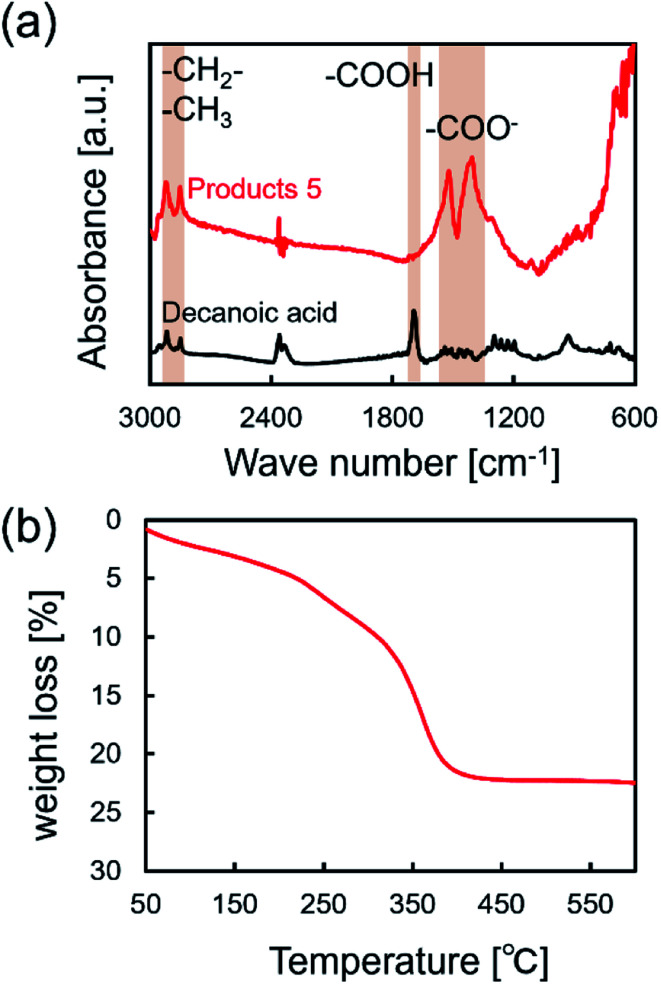
(a) FT-IR spectra of NCs synthesized in supercritical CO_2_ with decanoic acid (products 5 listed in Table 1) and pure decanoic acid. (b) TG spectra of NCs synthesized in supercritical CO_2_ with decanoic acid.

**Fig. 5:**
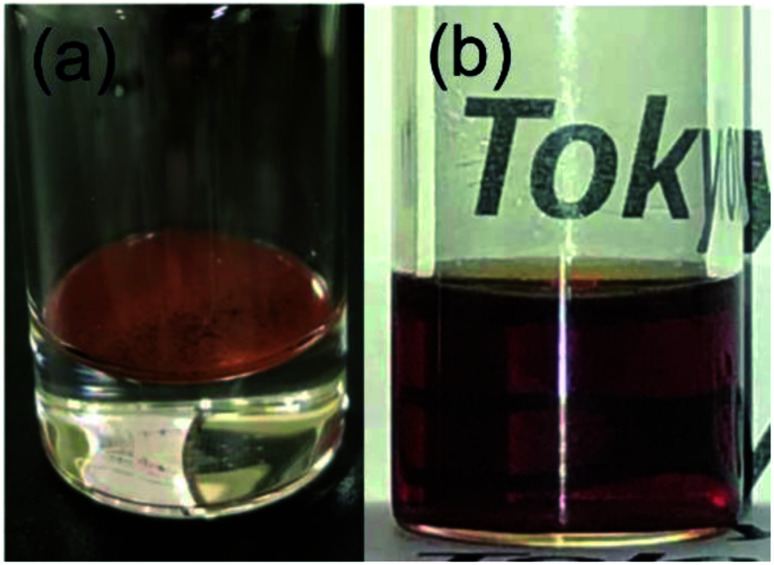
The optical images of the obtained NCs (products 5 listed in Table 1) with a concentration of 0.2% (w/v) in (a) water and (b) cyclohexane.

FT-IR and TG analyses were applied to characterize the surfactant attached on the surface of NCs synthesized in supercritical CO_2_ with decanoic acid. Fig. 4a shows the FT-IR spectra of the obtained NCs and pure decanoic acid. The characteristic bands at 2850 and 2900 cm^−1^ were assigned to asymmetric and symmetric stretching modes of –CH_2_– in the alkyl chains of monocarboxylic acid.^32^ However, the band assigned to the free carboxyl group (–COOH) of monocarboxylic acid was not observed, where its band is normally detected at about 1700 cm^−1^.^32^ Furthermore, bands at approximately 1530 and 1400 cm^−1^ can be assigned to the asymmetric and symmetric stretching modes of the carboxylate group (–COO^−^) of monocarboxylic acid.^32^ These results evidentially show that decanoic acid does not physically adsorb on the surface but chemically attaches to the surface of iron oxide NCs.^20^Fig. 4b shows the TGA results for the obtained NCs. Increase in the weight loss accelerated from approximately 300 °C, which also supports that decanoic acid chemically attached to the surface of iron oxide NCs because increase in the weight loss should be stopped near the boiling point of decanoic acid (corresponding to 243 °C) in the case of physical absorption.^33,34^ Herein, chemically bonded dense surfactants on the surface typically enables good dispersion of NCs in organic solvents, which are essential for the self-assembly on the substrate.^19^ In this study, we could observe well dispersed NCs with a concentration of 0.2% (w/v) in cyclohexane, as shown in Fig. 5b. It was also confirmed that the NCs were self-assembled on the TEM substrate shown in Fig. 2b and c by dripping the obtained NCs dispersed in cyclohexane onto the TEM grid. These results reversely support that the obtained NCs is densely modified by the surfactant, which allow some practical applications such as the patterning and thin film fabrication.

## 4. Conclusions

In this study, we aimed to synthesize the surface modified iron oxide nanocrystals (NCs) in supercritical CO_2_ to demonstrate the feasibility of supercritical CO_2_ as the reaction field for the synthesis of them. To investigate the effects of supercritical CO_2_, the synthesis was performed at N_2_ atmosphere, at 30.0 ± 0.3 MPa of N_2_ and at 30.0 ± 0.8 MPa of CO_2_, where iron(iii) acetylacetonate, pure water and decanoic acid were used as starting materials. As a result, the yield of the solid materials significantly increased by using supercritical CO_2_ compared with the case using N_2_. Moreover, the addition of decanoic acid to supercritical CO_2_ was revealed to drastically inhibit the aggregation of NCs, which allowed the formation of single-nano sized crystals with the unimodal size distribution. Furthermore, FT-IR and TG analyses support the decanoic acid chemically attach to the surface of the NCs that are dispersed to cyclohexane. These results suggest that supercritical CO_2_ medium can be a new appealing reaction field to fabricate the densely modified NCs without liquid waste.

## Conflicts of interest

The authors declare no competing financial interest.

## Supplementary Material
